# Newborn hearing screening programme in Belgium: a consensus recommendation on risk factors

**DOI:** 10.1186/s12887-015-0479-4

**Published:** 2015-10-16

**Authors:** Bénédicte Vos, Christelle Senterre, Raphaël Lagasse, SurdiScreen Group, Alain Levêque

**Affiliations:** 1 Research Center Epidemiology, Biostatistics and Clinical Research, Université libre de Bruxelles (ULB), School of Public Health, Route de Lennik 808, Brussels, 1070 Belgium; 2 Research Center Health Policy and Systems – International Health, Université libre de Bruxelles (ULB), School of Public Health, Route de Lennik 808, Brussels, 1070 Belgium; 3 Centre d’Epidémiologie Périnatale (CEpiP), Route de Lennik 808, Brussels, 1070 Belgium

**Keywords:** Neonate, Risk factor, Screening, Hearing loss, GRADE, Consensus method

## Abstract

**Background:**

Understanding the risk factors for hearing loss is essential for designing the Belgian newborn hearing screening programme. Accordingly, they needed to be updated in accordance with current scientific knowledge. This study aimed to update the recommendations for the clinical management and follow-up of newborns with neonatal risk factors of hearing loss for the newborn screening programme in Belgium.

**Methods:**

A literature review was performed, and the Grading of Recommendations, Assessment, Development and Evaluation (GRADE) system assessment method was used to determine the level of evidence quality and strength of the recommendation for each risk factor. The state of scientific knowledge, levels of evidence quality, and graded recommendations were subsequently assessed using a three-round Delphi consensus process (two online questionnaires and one face-to-face meeting).

**Results:**

Congenital infections (i.e., cytomegalovirus, toxoplasmosis, and syphilis), a family history of hearing loss, consanguinity in (grand)parents, malformation syndromes, and foetal alcohol syndrome presented a ‘high’ level of evidence quality as neonatal risk factors for hearing loss. Because of the sensitivity of auditory function to bilirubin toxicity, hyperbilirubinaemia was assessed at a ‘moderate’ level of evidence quality. In contrast, a very low birth weight, low Apgar score, and hospitalisation in the neonatal intensive care unit ranged from ‘very low’ to ‘low’ levels, and ototoxic drugs were evidenced as ‘very low’. Possible explanations for these ‘very low’ and ‘low’ levels include the improved management of these health conditions or treatments, and methodological weaknesses such as confounding effects, which make it difficult to conclude on individual risk factors. In the recommendation statements, the experts emphasised avoiding unidentified neonatal hearing loss and opted to include risk factors for hearing loss even in cases with weak evidence. The panel also highlighted the cumulative effect of risk factors for hearing loss.

**Conclusions:**

We revised the recommendations for the clinical management and follow-up of newborns exhibiting neonatal risk factors for hearing loss on the basis of the aforementioned evidence-based approach and clinical experience from experts. The next step is the implementation of these findings in the Belgian screening programme.

**Electronic supplementary material:**

The online version of this article (doi:10.1186/s12887-015-0479-4) contains supplementary material, which is available to authorized users.

## Background

The prevalence of bilateral hearing loss is substantial, particularly in neonates admitted to the neonatal intensive care unit (NICU) who frequently present with risk factors for hearing loss. The prevalence of significant bilateral hearing loss in this group is 1–3 %, which is 10 times higher than that in the well-baby nursery population [1]. Furthermore, early intervention in hearing-impaired children (aged 6 months or earlier) improved their language and speech outcomes as well as their socio-emotional development [2–4]. Therefore, universal newborn hearing screening is widely recommended [5–7] and implemented by governments or mother and child health agencies.

Follow-up on toddlers’ hearing to diagnose potential delayed-onset or progressive hearing loss in childhood is a major issue. In 2007, the Joint Committee on Infant Hearing (JCIH) released a unique list of risk indicators associated with congenital/neonatal hearing loss and delayed-onset/acquired or progressive hearing loss [6]. The JCIH recommends monitoring hearing, and speech and language skills of all infants as well as performing an audiological assessment at least once by 24–30 months of age in infants presenting with one or more risk indicators from this list. Most newborn hearing screening programmes and other recommendation statements refer to the statements of the JCIH. However, some authors have recently highlighted that the literature does not corroborate some risk indicators listed by the JCIH, especially with respect to their relationship with postnatal hearing loss [8, 9].

As in other regions, in Belgium, knowing the risk factors for hearing loss is essential for designing a newborn hearing screening programme with different organisations and tests. According to the programme of the Fédération Wallonie-Bruxelles (FWB, the French-speaking area of Belgium) launched in 2006, different protocols and neonatal hearing tests are performed depending on the presence or absence of particular risk factors; in their absence, an automated screening test of the cochlea is performed, whereas an audiological assessment is recommended in the presence of risk factor(s). This audiological assessment comprises diagnostic tests that evaluate the entire auditory function, including that of the central auditory system. The identification of risk factors directs neonates to the appropriate clinical pathway and thus is essential.

Since the beginning of the newborn hearing screening programme in the FWB, the risk factors were based on the JCIH 2000 Position Statement [10] and the clinical experience of professionals from the FWB. However, this list of risk factors must be updated. Clinicians, specifically otorhinolaryngologists and paediatricians, initially requested this update because the removal, addition, and/or clarification of some risk factors were required in their clinical practice. New scientific findings and studies were subsequently published, leading to the updated JCIH Position Statement in 2007 [6].

The present study aimed to update the recommendation for the clinical management of newborns with neonatal risk factors for hearing loss on the basis of current scientific knowledge. The recommendations were obtained by performing a literature review and then grading the evidence. Finally, the recommendations were validated by the consensus of a panel of experts in the context of the newborn hearing screening programme in the FWB. We also present the recommended follow-up regime for newborns with neonatal risk factors for hearing loss.

## Methods

**Fig. 1:**
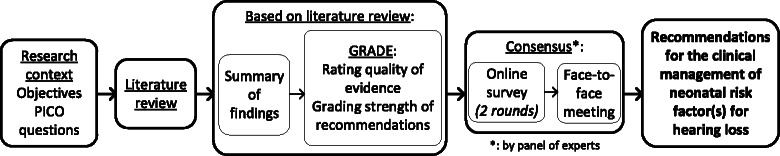
Flowchart of the methodological process

A consensus research procedure was used to update the clinical management of newborns exhibiting neonatal risk factors for hearing loss in the newborn hearing screening programme in the FWB (Fig. 1).

### Research context

To define the research context, objectives and research questions were clarified using the population, intervention, comparison, and outcomes (PICO) tool. This framework was applied to each risk factor for hearing loss used in the newborn hearing screening programme in the FWB (Table 1).

**Table 1:** Original neonatal risk factors for hearing loss in the newborn hearing screening programme

Congenital infections:
In utero infection due to cytomegalovirus, toxoplasmosis, herpes, rubella, and syphilis
Genetics of hearing loss:
Family history of hereditary hearing loss
Consanguinity in the first degree (i.e., parents are cousins)
Head or neck malformations, and by extension each polymalformation syndrome known to include hearing loss
Maternal intoxication during pregnancy:
Poisoning (alcohol or drugs) by the mother during pregnancy
Specific conditions of the neonate:
Gestational age <36 weeks and/or birth weight <1,500 g
Apgar score of 0–6 at 5 min
Exchange transfusion (see reference curves) (hyperbilirubinaemia or Rhesus incompatibility)
Medical care:
Neonatal intensive care unit stay >5 days
Newborn ototoxic medication
Assisted ventilation ≥24 h
Particular diseases:
Neurologic disease of the newborn (e.g., meningitis, etc.)
Endocrine disease of the newborn (e.g., thyroidal disease, etc.)

### Literature review

Between September 2014 and December 2014, we reviewed the literature from the last 15 years for each risk factor on the original list, and aimed to answer two specific questions for each risk factor: (1) is it scientifically pertinent to consider it as a risk factor for hearing loss in the newborn hearing screening programme? and (2) how is the risk factor defined?

We reviewed the PubMed database, the academic library of our institution, and the Cochrane Library for articles in English and French. The following search terms were used: [‘hearing loss’ OR ‘hearing impairment’ OR ‘deafness’] AND [‘newborn’ OR ‘neonatal’]. In addition, each risk factor was searched (using Medical Subject Headings (MeSH) terms or not). To the extent possible, the literature review was limited to the last 15 years to avoid the effects of changes in healthcare. Nonetheless, if the literature review results were insufficient, the search period was prolonged and the literature research was extended to ‘neurodevelopmental outcomes’ on the condition that the articles in question investigated hearing loss. In cases in which few relevant papers were found, bibliographies were used to find other references. Review articles were included, but animal model studies were excluded. The literature review revealed three potential risk factors not included in the original list that were included in the analysis: congenital diaphragmatic hernia, extracorporeal membrane oxygenation, and inhaled nitric oxide.

When available in the selected papers, scientific information about follow-up and postnatal hearing loss was also reviewed for the risk factors from the original list (i.e., what kinds of tests and timing are necessary?). To ensure all risk factors were included in the list of the FWB, a global literature review was performed using the search terms ‘neonatal hearing loss’ and ‘postnatal hearing loss’. We also searched the web to identify specific documents from other newborn hearing screening programmes (i.e., grey literature).

### Level of evidence

The main findings from each paper were summarised in a table using the same framework for all risk factors. The state of the scientific knowledge was subsequently rated according to the Grading of Recommendations, Assessment, Development and Evaluation (GRADE) system [11]. The quality of evidence of each risk factor from the screening programme as a risk factor for hearing loss was rated on the basis of the summary of scientific literature. The level of quality was rated as high, moderate, low, or very low. Considering the PICO questions and study objectives, the studies reviewed were mostly observational, starting at a ‘low’ level of quality. Nevertheless, the rating can be upgraded or downgraded according to the methodological elements of the studies [12, 13].

### Strength of the recommendations

Recommendations were formulated on the basis of the literature review, quality of evidence, confidence in the ‘balance between desirable and undesirable effects’ of the management strategies, required resources, and patients’ values and preferences (based on experts’ experience) [14]. Each recommendation was graded as ‘recommend’ (strong) or ‘suggest’ (weak) [14]. Regarding the hearing screening programme, ‘audiological assessment’ referred to detailed hearing tests that should be implemented in the presence of risk factors for hearing loss. Conversely, ‘screening test’ referred to the mass screening test performed in the absence of a given neonatal risk factor.

### Consensus method

The Delphi consensus method was used. Experts in paediatrics, otorhinolaryngology, or newborn hearing screening were recruited to participate in the panel of experts. The final panel was comprised of six otorhinolaryngologists (from university or non-university hospitals or hearing rehabilitation centres), three paediatricians (from hospitals or the Mother and Child Health Agency), and one neurophysiologist. These experts were not mandated by a government organisation and had no competing interests related to this project; their sole motivation was to improve the newborn hearing screening programme in accordance with clinical realities and current medical knowledge.

The consensus process was conducted between January 2015 and April 2015 in three rounds: two rounds of online questionnaires and one face-to-face meeting. The first online questionnaire aimed to validate the state of scientific knowledge, the rated evidence for each neonatal risk factor, and the proposed graded recommendations. All pertinent scientific literature and the aforementioned summary of the scientific papers were available for the panel of experts. The online surveys were conducted using SurveyMonkey®, and responses were coded on a Likert scale from 1 to 5 (strongly agree, agree, neutral, disagree, and strongly disagree, respectively). A consensus was reached when at least two-thirds of the panel agreed or disagreed, and when the mean Likert score was ≤2 or ≥4 [15]. A second questionnaire was administered for discordant items related to recommendations; non-consensual states of scientific knowledge and rated evidence were adapted. The second online questionnaire used stricter criteria with a narrow Likert scale from 1 to 3 (agree, neutral, and disagree, respectively), or only two answer options were provided. The advantage of the online survey was that each expert had the opportunity to independently express his/her opinion. Finally, the panel of experts discussed persistent discordances from the second online questionnaire during the face-to-face meeting to reach a final consensus.

### Ethical concern

The ethical approval and informed consent are not necessary according to Belgian regulations (the clinical research Act - 2004 and the privacy Act - 1998).

## Results

The rated levels of evidence and graded recommendations for each risk factor are summarised in Table 2.

**Table 2:** Level of the quality of evidence and strength of the recommendation for each risk factor

Risk factor	Quality of evidence	Strength of recommendation
Congenital cytomegalovirus	High	Strong
Congenital toxoplasmosis	High	Strong
Congenital syphilis	High	Strong
Congenital rubella	High	Strong
Congenital herpes	Very low	Strong^a^
Family history of hearing loss	Moderate	Strong
Consanguinity	Moderate	Strong
Malformations and syndromes associated with hearing loss	High	Strong
Malformations of the pinnae (isolated)	Low	Weak
Maternal intoxication: foetal alcohol syndrome	Moderate	Strong
Maternal intoxication: drug abuse	Very low	Strong^a^
Very low birth weight	Very low	Strong
Birth asphyxia/Apgar score	Low	Strong
Hyperbilirubinaemia	Moderate	Strong
Neonatal intensive care unit stay	Very low	Weak
Assisted ventilation	Very low	Weak
Ototoxic drugs: aminoglycosides	Very low	Weak
Ototoxic drugs: loop diuretics	Very low	Weak
Extracorporeal membrane oxygenation	Moderate	Strong
Congenital diaphragmatic hernia	Very low	--^b^
Inhaled nitric oxide	Very low	--^b^
Neurologic disease: meningitis	Moderate	Strong^c^
Neurologic disease: intraventricular haemorrhage	Very low	Weak
Congenital hypothyroidism	Moderate	Strong

^a^Strongly recommended to **not** consider this as a risk factor for hearing loss

^b^The panel decided to formulate no specific recommendation; because of the medical condition, ventilation will be performed (see recommendation for assisted ventilation)

^c^On the basis of their clinical experience, the panel decided to recommend an audiological assessment for newborns who need a neurologic consultation (e.g., convulsion, hypotonia, swallowing/feeding difficulties, or cranial nerve palsy)

### Congenital infections

The prevalence of sensorineural hearing loss was higher in neonates with congenital cytomegalovirus (CMV) than those without risk factors for hearing impairment. Observational studies (i.e., cohort and case–control studies) reported a strong association in neonates between hearing impairment and congenital CMV infection regardless of whether the infection is symptomatic or asymptomatic at birth [16, 17]. Moreover, studies following children during infancy also reported late-onset or fluctuating hearing loss, highlighting the need for audiological follow-up of infants with congenital CMV during childhood [16–18]. Antiviral therapy in neonates with congenital CMV has improved developmental and hearing outcomes but has not resulted in total recovery [19–21]. Antiviral therapy is recommended for symptomatic neonates but not all congenital CMV-infected neonates.

Similarly, congenital toxoplasmosis, syphilis, and rubella are reportedly associated with neonatal hearing impairment [22–27]. Nowadays, treatments for congenital toxoplasmosis and syphilis, and rubella vaccine are administered. Current results show no evidence for the association between sensorineural hearing loss in neonates with congenital toxoplasmosis or syphilis when adequately treated [25, 28–31]. Nevertheless, follow-up evaluation of hearing is recommended even for adequately treated cases of toxoplasmosis or syphilis [25, 29]. Furthermore, infants with congenital rubella syndrome have an elevated risk of hearing impairment. Therefore, neonatal hearing evaluation and follow-up during childhood are required for rubella-infected newborns [27, 32]. Nonetheless, widespread rubella vaccination has dramatically reduced the incidence of the disease. Consequently, hearing impairment due to congenital rubella is now rare. However, the small number of cases is an issue in some studies, resulting in low statistical power.

A systematic literature review evaluated the association between congenital herpes infections and sensorineural hearing loss in neonates: only three studies were identified, and limited evidence supports the assumption that herpes simplex virus infection is a cause of sensorineural hearing loss [33]. Methodological limitations such as inaccurate audiological information and the timing of infection, and the imprecise timing of tests limit the strength of this association.

#### Recommendations

The panel of experts recommends performing audiological assessment during the neonatal period for newborns with congenital CMV, toxoplasmosis, rubella (i.e., congenital rubella syndrome), or syphilis infection.

The panel of experts recommends performing a hearing screening test on newborns with congenital herpes simplex virus.

The panel of experts clarifies that congenital infection means that the newborn is infected, not merely maternal seroconversion during pregnancy. Neonatal infection should be identified by blood test (i.e., serologic confirmation of toxoplasmosis, rubella, and syphilis) or urine test (i.e., CMV infection) according to the infection.

This recommendation places high value on the level of evidence of the risk factor for hearing loss and low value on the treatment status of the neonate.

### Genetics of hearing loss: family history, consanguinity, syndromes, and malformations

In the literature, a family history of hearing loss is often analysed with consanguinity [34–36]. However, the state of genetics-related knowledge suggests a family history as a potential risk factor of hearing loss in infants [37–39], particularly postnatal hearing loss was recently demonstrated [40]. The literature clearly demonstrates that the degree of parental consanguinity is significantly and directly associated with the prevalence of hearing loss in children [34, 41–43]. Furthermore, knowledge on genetics also indicates that specific congenital syndromes are associated with hearing loss; multiple websites and reviews have taken inventory of such conditions [44–46]. Isolated malformations of the pinnae such as preauricular skin tags and/or ear pits are associated (significantly associated in two of three studies) with a higher prevalence of hearing impairment in most studies [47–49]. In addition, cleft palate is associated with an increased prevalence of conductive hearing loss in children even after surgical repair [50, 51].

#### Recommendations

The panel of experts recommends performing audiological assessment during the neonatal period in cases with (a) a family history of congenital or early-onset hereditary hearing loss (in parents, grandparents, siblings, or cousins); (b) consanguinity of first or second degree (i.e., parents or grandparents are cousins); and (c) malformations and syndromes associated with hearing loss.

The panel of experts suggests audiological assessment during the neonatal period in the presence of isolated malformations of the pinnae.

### Maternal intoxication during pregnancy

Maternal alcohol consumption in pregnancy, without foetal alcohol syndrome, is not a risk factor for hearing loss. Nevertheless, the prevalence of sensorineural and conductive hearing loss is higher among children who suffered from foetal alcohol syndrome than in the general paediatric population [52–54]; the rates of hearing defects were similar to those of newborns with craniofacial anomalies [55–57]. However, other maternal drug abuse during pregnancy, such as cocaine, heroin, and methadone, is not significantly associated with neonatal hearing impairment; studies either failed to show evidence of a significant association or the effects of these drugs on auditory function were inconsistent among studies [57–61].

#### Recommendations

The panel of experts recommends performing audiological assessment during the neonatal period in the presence of foetal alcohol syndrome.

The panel of experts recommends performing a hearing screening test in neonates born to mothers who abused drugs during pregnancy.

### Specific neonatal conditions: prematurity or low birth weight, Apgar scores, and hyperbilirubinaemia

Studies analysing low birth weight used different classifications of birth weight, such as low, very low, or extremely low birth weight. Most studies do not provide evidence of a direct association between the neonatal hearing loss and low birth weight, although the prevalence of sensorineural hearing loss is higher in low-birth-weight neonates [62–66]. This can be explained by the factors commonly related to low birth weight that may have impacted hearing, such as assisted ventilation, ototoxic drug administration, or hyperbilirubinaemia [67, 68]. Most studies failed to account for these confounding variables in multivariable analysis. Therefore, it weakens the strength of the association.

Another specific indicator of neonates is the Apgar score, which is used as an indicator of birth asphyxia. Studies analysing the association between Apgar score with hearing loss were difficult to compare: the timing of the Apgar score (i.e., 1, 5, or 10 min after birth) and cut-off for birth asphyxia (i.e., Apgar score <3, ≤6 or <6, ≤7 or <7, etc.) varied considerably. In some studies, the Apgar score was not associated with hearing loss, whereas in others, a low Apgar score was associated with sensorineural hearing loss or abnormal hearing results, particularly when measured 5 min after birth (i.e., scores <3 or ≤6, or ≤7) [69–73]. Therefore, further studies are required to clarify the duration of asphyxia, permanent characteristics of hearing deficits related to the Apgar score and birth asphyxia, and role of prematurity, which appears to be a confounding factor [69, 74].

Hyperbilirubinaemia is frequently encountered in neonates; severe and very severe cases must be treated by phototherapy or exchange transfusion, respectively. Hearing disabilities among infants with a history of hyperbilirubinaemia are more prevalent than in the general paediatric population [75, 76]. Indeed, the auditory system is sensitive to bilirubin toxicity, which may lead to bilirubin-induced neurologic dysfunction (BIND) syndrome [77–82]. Some factors such as prematurity, sepsis, and hypoxia may exacerbate bilirubin toxicity [79, 80, 83, 84]. The most frequent type of auditory damage is auditory neuropathy or dyssynchrony [80–82]. However, some hearing disabilities are transient and improve with a decrease in the bilirubin level [85, 86]. Among preterm and full-term infants, the total serum bilirubin level does not appear to be a sensitive or specific indicator for assessing the risk of auditory damage [83]. Moreover, auditory impairment may occur at total bilirubin levels considered ‘safe’ [80, 84]. Several studies mentioned that besides the bilirubin level, the duration of exposure to bilirubin is related to hearing loss [80, 81, 83]. Therefore, risk assessment for auditory impairment in cases of hyperbilirubinaemia should include several biomarkers and auditory tests [80, 82, 84].

Very low birth weight/prematurity, a low Apgar score/birth asphyxia, and hyperbilirubinaemia have a cumulative effect, increasing the vulnerability of the brain and auditory function [69, 74, 76, 78, 84].

#### Recommendations

The panel of experts recommends performing audiological assessment during the neonatal period in cases of (a) a very low birth weight (<1,500 g); (b) an Apgar score of 0–6 at 5 min; and (c) early hyperbilirubinaemia (before day 2) requiring treatment or hyperbilirubinaemia at any day of life requiring either intensive phototherapy or exchange transfusion (based on reference curves).

By placing a high value on avoiding unidentified neonatal hearing loss and because it is painless to perform an audiological assessment, the panel of experts considers very low birth weight a risk factor for hearing loss, even with a ‘very low’ level of evidence. Moreover, in addition to exchange transfusion, the panel considers early hyperbilirubinaemia and intensive phototherapy a stronger risk factor than that in the JCIH Position Statement (2007) [6]. The panel stresses that improved phototherapy techniques and devices lead to less frequent exchange transfusion treatments. Therefore, they recommend considering early hyperbilirubinaemia and intensive phototherapy as neonatal risk factors for hearing loss; they specifically choose not to make a recommendation based on clinical markers. From a broader perspective, the panel decided a strong recommendation for these three conditions because of their cumulative impact on auditory function susceptibility.

### Medical care: NICU stay and use of ventilation or ototoxic drugs

The JCIH Position Statement (2007) [6] considers a NICU stay exceeding 5 days to be risk factor associated with permanent congenital, delayed, or progressive hearing loss [6]. The association between NICU stay (i.e., admission or length of stay) and hearing loss is controversial [73, 87–89]. Indeed, this indicator encompasses multiple conditions and treatments and thus may not reflect the complex and variable health situation of neonates hospitalised in the NICU. Moreover, this indicator is insufficiently considered by multivariable statistical models.

Newborns admitted to the NICU can receive ventilation support with endotracheal ventilation or nasal continuous positive airway pressure (CPAP). The prevalence of hearing loss does not differ significantly between mechanical ventilation and CPAP [90, 91]. Multivariable analyses performed exclusively on preterm neonates indicate that assisted ventilation lasting >5 days is an independent risk factor for hearing loss and a risk of failed hearing screening tests [74, 90]. However, a study of newborns admitted to the NICU reported no significant association between hearing loss and endotracheal assisted ventilation or CPAP after adjusting for infants’ characteristics and specialised medical procedures [73]. Univariate analyses of different studies of newborns admitted to the NICU showed no significant association between hearing loss and assisted ventilation regardless of whether treatment duration was mentioned. Therefore, current evidence indicates that assisted ventilation is not obviously a neonatal risk factor for hearing loss [92, 93].

Ototoxic drugs, specifically aminoglycosides and loop diuretics, can be administered to newborns. However, the association between aminoglycoside administration and hearing loss is inconsistent among studies; most studies reported no significant association with treatment duration, total dose, or peak or trough serum concentrations [94–98], whereas others reported ototoxicity of aminoglycosides [88, 94, 95, 97–100], particularly on high-frequency hearing [67, 99]. In some individuals, genetic predisposition (i.e., a specific mutation of mitochondrial DNA) is associated with aminoglycoside-induced and non-syndromic sensorineural hearing loss, making them particularly vulnerable to aminoglycoside toxicity [101]. The association between loop diuretics administered to neonates and hearing loss is also inconsistent. However, their (over) use in combination with other treatments (e.g., aminoglycosides) appears to be associated with sensorineural hearing loss [97, 102]. The transient characteristic of loop diuretic-associated hearing loss has also been discussed [67]. In those studies, the administration of ototoxic drugs was supposed to be clinically appropriate; inappropriate or uncontrolled drug administration may have shown a different association with hearing loss.

Extracorporeal membrane oxygenation (ECMO) is an extreme medical therapy used in critically ill newborns. The incidence of sensorineural hearing loss reported among infants who have received ECMO varies widely among studies, but is higher than that in the general paediatric population [103, 104]. However, these neonates also received other extreme treatments and medical care that could be related to sensorineural hearing loss. As the hearing impairment reported in ECMO-treated neonates may be late-onset or progressive, follow-up during childhood is recommended among those treated with ECMO [103–105]. Studies about hearing loss frequently investigated congenital diaphragmatic hernia and inhaled nitric oxide in combination with ECMO. The incidence rates of hearing loss associated with congenital diaphragmatic hernia are inconsistent in the literature [106–109]. Moreover, such infants require other treatments and may suffer from other conditions that are associated with or are considered risk factors for sensorineural hearing loss. Furthermore, there is no significant difference in the rate of sensorineural hearing loss between neonates treated with inhaled nitric oxide and those treated with either 100 % oxygen or simulated initiation treatment [110, 111]. The high prevalence of sensorineural hearing loss in neonates treated with inhaled nitric oxide may be due to other conditions or therapies. Hence, the relationship between hearing loss and specific conditions and treatments in critically ill neonates, such as ECMO, congenital diaphragmatic hernia, and inhaled nitric oxide, require further investigation despite the small numbers of cases.

#### Recommendations

The panel of experts recommends performing audiological assessment during the neonatal period after ECMO treatment.

The panel of experts suggests performing audiological assessment during the neonatal period in cases of (a) a NICU stay exceeding 5 days; (b) assisted ventilation lasting at least 24 h; and (c) ototoxic drug (i.e., aminoglycosides or loop diuretics) administration regardless of treatment length.

The panel of experts’ recommendations regarding NICU stay and ototoxic drugs are concordant with those of the JCIH Position Statement (2007) [6]. They consider assisted ventilation to include mechanical ventilation (with endotracheal intubation) and CPAP. The 24-h duration of assisted ventilation was included as a criterion of ill newborns. The panel proposed no specific recommendations regarding neonates suffering from congenital diaphragmatic hernia or those treated with inhaled nitric oxide; because of their medical condition, they will be ventilated and thus should have an audiological assessment as suggested.

### Specific diseases: neurologic or endocrine diseases

Neurologic diseases in neonates include meningitis and intraventricular haemorrhage. The risk of hearing loss due to meningitis varies widely in the literature, although the reported rates are higher than those in the general population; the long-term consequences such as improvement/worsening of impairment also vary [112–114]. Neonates with intraventricular haemorrhage, which is specific to preterm infants, exhibit a slightly higher prevalence of hearing loss, but an in-depth study highlights the role of white matter lesions over the intraventricular haemorrhage on neurodevelopmental outcomes such hearing loss [115–117].

Congenital hypothyroidism is strongly associated with a higher prevalence of hearing loss than that in the general population; reported cases of hearing loss are mostly bilateral and of mild to moderate severity [118–120]. Phenylketonuria is a congenital endocrine disease that is universally screened and treated; therefore, an association between this disease, specifically if untreated, and hearing loss is difficult to determine; the associations of other endocrine diseases such the thrifty phenotype hypothesis with hearing loss also require further investigation [121, 122].

#### Recommendations

The panel of experts recommends performing audiological assessment during the neonatal period in neonates (a) who have suffered from meningitis or require a neurologic consultation (i.e., convulsion, hypotonia, swallowing/feeding difficulties, and cranial nerve palsy) and (b) with congenital hypothyroidism.

Meningitis is confirmed by positive culture. The panel of experts highlights some specific neurologic conditions for paediatricians and otorhinolaryngologists, even without rigorous evidence of an association with hearing loss. This recommendation is based on the experts’ clinical experience.

The panel of experts suggests performing audiological assessment during the neonatal period in neonates with white matter lesions or intraventricular haemorrhage.

### Specific elements emerging from the consensus

#### Cumulative effect of risk factors on hearing function

The panel of experts explicitly highlights situations in which newborns exhibit more than one risk factor for hearing loss; the prevalence and severity of hearing loss increase with an increasing number of risk factors [123]. Therefore, such newborns require special attention.

#### Reassessment of risk factors

The panel insists these neonatal risk factors for hearing loss are applicable during the first month of life and should be reassessed in cases showing changes in health condition during that period (e.g., in case of readmission).

### Recommended hearing tests and timing of the tests (initial assessment and follow-up)

#### Timing of the initial audiological assessment

When an audiological assessment is suggested or recommended, the panel of experts highlights the necessity to perform (to the extent possible) hearing tests before hospital discharge. In cases involving admission to the NICU in particular, neonate hospitalisation is stressful for the parents; therefore, they should not be required to return to the hospital after discharge, if possible. The goal is to avoid losses to follow-up and thus undiagnosed cases. If audiological assessment is not performed before discharge because of a short hospital stay, an outpatient appointment should be made during the following month at the latest. The appointment should be scheduled before discharge.

#### Hearing tests for the initial audiological assessment

The panel of experts states that the audiological assessment should at least include an auditory brainstem response to assess the entire auditory brainstem pathway. The tests should be chosen within the competency of the otorhinolaryngologist in charge of the patient and in accordance with the context and situation.

#### Follow-up

The panel of experts recommends audiological follow-up for all children who have undergone audiological assessment at birth; this follow-up should be performed once between the ninth and twelfth months of life. The panel of experts identifies two exceptions. First, children with congenital CMV infection, a family history of congenital or early-onset hereditary hearing loss, a family history of consanguinity of the first or second degree, malformations and syndromes associated with hearing loss, or those treated with ECMO should undergo audiological follow-up every 4–6 months during their first two years of life. Their hearing should be reassessed annually between 2 and 6 years of age. Second, neonates treated with ototoxic drugs should undergo audiological follow-up once during the first 3 months of life. The otorhinolaryngologist will judge the appropriate hearing tests to perform during follow-up, depending on the child’s risk factors and age.

## Discussion

This study aimed to update the recommendations for the clinical management and follow-up of children with neonatal risk factors for hearing loss in the newborn hearing screening programme in the FWB in Belgium. To this end, we used methodological tools, including the formulation of PICO questions, a literature review to establish the state of scientific knowledge on risk factors for hearing loss, the GRADE system assessment, and a consensus process with a panel of experts. The findings of this study will ultimately improve clinical practice through the earlier identification of newborns suffering from hearing loss and adequate follow-up of children at risk of delayed or late-onset hearing loss. Indeed, the newborn hearing screening programme in Belgium is based on the presence or absence of risk factor(s) for hearing loss. Therefore, an accurate, sensitive, and well-formulated list of neonatal risk factors for hearing loss and recommendations for their management is essential. However, the misidentification of risk factors for hearing loss may lead to unnecessary assessment and stress for parents; alternatively, newborns with neonatal hearing loss may be overlooked because of having been subjected to an insufficiently accurate audiological test. Modification of the protocol design of the hearing screening programme was unfeasible because it would have required the analysis of complex technical questions such as available automated hearing tests and their classification algorithms of normal versus unsatisfactory results, organisational matters, cost-effectiveness, and the global system of newborn hearing screening in Belgium.

The state of knowledge about the neonatal risk factors for hearing loss highlights the effects of treatment; in the cases of some infectious risk factors such as congenital CMV, toxoplasmosis, and syphilis infections, treatments have modified the risk of developing hearing loss or prevented hearing deterioration. When such diseases are treated early after birth, congenitally infected children have a lower risk of developing hearing loss than those without treatment [20, 25, 29]. Therefore, the early identification of these risk factors is essential during prenatal care or at birth at the latest to initiate treatment. The rubella vaccination already changed the situation; because of the vaccine and widespread immunization, congenital rubella and hearing loss due to the disease have become extremely rare in Belgium [27]. However, congenital rubella must still be considered a risk factor in the hearing screening programme, particularly for neonates born to unvaccinated mothers. Likewise, the treatment of hyperbilirubinaemia reduces the risk of auditory damage. Treatment reduces the risk of developing hearing loss in some cases, whereas treatments such as ototoxic drugs, ECMO, or ventilation are actually risk factors. Although the development and evolution of these techniques or treatments has led to better control, they must still be used carefully and newborns should be monitored closely. Because of these advances in healthcare, analysing risk factors for hearing loss on the basis of studies performed decades ago is not recommended. Therefore, to avoid inaccurate information, we limited our literature review as much as possible to articles published during the last 15 years. We also rated the quality of evidence without including the treatment effect to ensure a standardised perspective.

Drawing conclusions from published studies was sometimes made difficult by the studies themselves. In particular, multivariable analyses were not performed systematically, and the numbers of children with hearing impairment identified in the studies were limited. Firstly, ill newborns frequently exhibit multiple risk factors such as prematurity, NICU stay, ototoxic drug administration, ventilation, etc. Hence, it is essential to consider the actual impact of individual risk factors on hearing function. Therefore, univariate analyses were insufficient, and multivariable models were not performed systematically. Second, the few cases of children with hearing impairment also complicated the drawing of conclusions; the low prevalence (a small percentage) of hearing loss applied to small samples (usually <500 newborns) led to the identification of only a few hearing-impaired newborns. Random sampling may have dramatically affected the numbers of identified children with hearing impairment results and thus statistical power. Furthermore, there were multiple definitions of hearing loss and a wide range of hearing tests and clinical criteria as well as failure to consider other risk factors and inconsistent results among studies in some cases.

To avoid bias in the literature review and determine the states of scientific knowledge, we developed a thorough and exhaustive approach. Although we investigated the risk factors for hearing loss individually, when pertinent, we presented their cumulative effects on hearing function by using a transversal approach. By making specific inquiries during the literature review, we detected risk factors for hearing loss that would not have been included in the original list. Because of this exhaustive approach, we decided to add three risk factors to our investigation: ECMO, congenital diaphragmatic hernia, and inhaled nitric oxide. We strictly limited our research to medical conditions and factors, although sociodemographic factors have been reported as other potential risk factors for neonatal hearing loss [124]. Although the association of sociodemographic factors with hearing loss is poorly understood, they appear to be part of a more complex relationship; that is, sociodemographic factors appeared to be related to medical conditions or risk factors already associated with hearing loss. These kinds of risk factors were not included in the newborn hearing screening programme but should be monitored in the FWB to clarify their associations.

It is important to note that the studies retrieved through the literature review were mostly observational and thus started at a ‘low’ level of evidence quality. Nonetheless, the GRADE system is flexible, as the quality of evidence can be rated on the basis of methodological criteria [125]. Indeed, we uprated the quality of evidence for some risk factors even though the studies were exclusively observational [11]. However, rating the quality of evidence involves making a judgment to choose the best classification, whereas quality generally appears to be distributed in a continuum; this arbitrariness implies subjectivity. The transparency of the process and the detailed summary of findings and arguments that arose when rating the levels of evidence helped reduce this subjectivity. With the aim of transparency, we mentioned the arguments for the levels of evidence quality (Additional file 1: Table S1).

The GRADE system is usually used in the clinical management of therapies but can also be used in all healthcare management decision making [13, 126]. Our research objective did not focus on therapy or diagnostic tests [127]. However, the Belgian Health Care Knowledge Centre used the GRADE system to identify risk factors for a breast screening programme [128]. This bolsters our confidence in the application of the GRADE system in the present study. Nonetheless, the panel of experts was not always comfortable with the application of the GRADE system. They were all clinicians, and the conflict between the evidence-based (i.e., epidemiological) approach and their clinical experience (i.e., individual approach) was challenging. Rating the quality of evidence needed to be discussed specifically during the face-to-face meeting; in particular, it was pointed out that a low quality of evidence does not necessarily mean that the element should not be considered a risk factor for hearing loss but that the rating also results from the type of study, biases, limitations, results, and methodology.

Beyond the quality of evidence, the recommendation formulation considers other parameters such as benefit/harm balance, resource use, and patients’ values and preferences [14]. The panel of experts aided the transition from quality of evidence to recommendations owing to their medical expertise and knowledge about the subject and clinical practice. Indeed, in our context, the inadequate identification of risk factors for hearing loss affected the balance between misdiagnosis (or delayed diagnosis), parental stress, and good allocation of human, technical, and financial resources (for the family and society). In the absence of known risk factors for hearing loss, a mass screening test is performed by professionals with basic training (i.e., professionals not specialised in audiology); this requires less time and a less expensive device, incurring less parental stress than an audiological assessment. However, the automated otoacoustic emissions hearing screening technique implemented in the screening programme is not sufficiently sensitive in the presence of risk factor(s) and may not identify neonatal hearing loss. Indeed, retrocochlear hearing loss is more frequently encountered in the presence of a risk factor and may not be detected by an otoacoustic emissions test (cochlear testing). Therefore, an up-to-date list of risk factors and recommendations is important to ensure the quality of the newborn hearing screening programme and the health system sector responsible for hearing problems. In cases in which it was unclear whether to include a risk factor in the updated list, the panel of experts gave more weight to the fact that audiological assessment is not painful and prevents delayed diagnosis, even though this technique requires more time for both the family and healthcare professionals and can incur parental stress; in such cases, they developed a conservative approach and always considered unclear factors as risk factors for hearing loss. In other words, the expert panel emphasises avoiding delayed diagnosis or misdiagnosis, even if incurring unnecessary hearing tests. According to the GRADE system, the ‘patient important outcome’ must be included in the recommendations [14]. Patients’ values and preferences must also be included in the grading [14]. However, during the entire research process, more precisely in the development of recommendations, patients’ or their parents’/guardians’ perspectives were not consulted; their input was only indirectly included according to the clinical experiences of the panel of experts.

To foster rapid and complete acceptance, the recommendations were written to be helpful and clear. The next step after the publication of these updated recommendations is to implement the updated list of risk factors for hearing loss in the programme. The challenge is to convince paediatricians and otorhinolaryngologists to implement this updated list and follow the recommendations by adapting their clinical practice. Having pertinent and adequate recommendations but not using them would negatively impact the newborn hearing screening programme. Therefore, the dissemination of the updated list, recommendations, and underlying scientific arguments is strongly advised. They can be presented at national paediatric or otorhinolaryngology congresses and directly to local hospital staff.

## Conclusions

We updated the risk factors for hearing loss for the newborn hearing screening programme in Belgium (FWB) by combining an evidence-based approach and the clinical experience of a panel of experts. Hence, we developed recommendations for the clinical management and follow-up of newborns with neonatal risk factors for hearing loss. As the recommended hearing tests and follow-up regime differ depending on the presence or absence of these risk factors in newborns, it is essential to correctly identify newborns with neonatal risk factor(s) for hearing loss. The quality of evidence for the risk factors for hearing loss ranged from ‘very low’, due to the absence of scientific evidence or methodological weaknesses in studies, to ‘high’, mostly when the physiopathology of the risk factor on the auditory function is understood. The recommendations were also graded as ‘weak’ or ‘strong’. Moreover, the panel of experts emphasises avoiding unidentified neonatal hearing loss and recommends considering unclear factors as risk factors for hearing loss. The next step is to implement these recommendations, which will improve the ability of the hearing screening programme to identify hearing loss in children and perform adequate follow-up of children at risk of later onset; regular monitoring of the screening programme should integrate this updated list of risk factors.

## Additional file

Additional file 1: Table S1. Rating of the quality of evidence for the risk factors for hearing loss. (PDF 51 kb)

## Abbreviations

BIND: Bilirubin-induced neurologic dysfunction
CMV: Cytomegalovirus
CPAP: Continuous positive airway pressure
ECMO: Extracorporeal membrane oxygenation
FWB: *Fédération Wallonie-Bruxelles*
GRADE: Grading of Recommendations, Assessment, Development and Evaluation
JCIH: Joint Committee on Infant Hearing
MeSH: Medical Subject Headings
NICU: Neonatal intensive care unit
PICO: Population, intervention, comparison, and outcomes
