# Supplementary material for: Newborn hearing screening programme in Belgium: a consensus recommendation on risk factors
Source: BMC Pediatr. 2015 Oct 16;15:160. doi: 10.1186/s12887-015-0479-4 (PMC4609128; doi:10.1186/s12887-015-0479-4)
Supplement: Additional file 1: Table S1. — Rating of the quality of evidence for the risk factors for hearing loss. (PDF 51 kb) [file 12887_2015_479_MOESM1_ESM.pdf]

Additional file

Table 1: Rating of the quality of evidence for the risk factors for hearing loss

	**Study limitations**	**Inconsistent results**	**Indirectness of evidence**	**Imprecision**	**Publication bias**	**Uprating**	**Quality of evidence**
Congenital cytomegalovirus	No serious limitations	No serious inconsistency	No serious indirectness	No serious imprecision	Undetected	Natural history known	High[1]
Congenital toxoplasmosis	No serious limitations	No serious inconsistency	No serious indirectness	No serious imprecision	Undetected	Natural history known	High
Congenital syphilis	No serious limitations	No serious inconsistency	No serious indirectness	No serious imprecision	Undetected	Natural history known[2]	High
Congenital rubella	No serious limitations	No serious inconsistency	No serious indirectness	No serious imprecision	Undetected	Natural history known; epidemiol. relation[3]	High
Congenital herpes	Serious limitations	Serious inconsistency	No serious indirectness	Serious imprecision	Undetected		Very low[4]
Family history of hearing loss	Serious limitations[5]	No serious inconsistency	Serious indirectness[6]	No serious imprecision	Undetected	Knowledge in genetics	Moderate[7]
Consanguinity	Serious limitations[5]	No serious inconsistency	Serious indirectness[6]	No serious imprecision	Undetected	Knowledge in genetics	Moderate[7]

Malformations and syndromes associated with hearing loss	No serious limitations	No serious inconsistency	No serious indirectness	No serious imprecision	Undetected	Knowledge in genetics	High[7]
Malformations of the pinnae (isolated)	No serious limitations	Serious inconsistency	No serious indirectness	No serious imprecision	Undetected		Low[8]
Maternal intoxication: foetal alcohol syndrome	No serious limitations	No serious inconsistency	No serious indirectness	No serious imprecision	Undetected	Very high prevalence of hearing loss	Moderate
Maternal intoxication: drug abuse	Serious limitations	Very serious inconsistency	No serious indirectness	No serious imprecision	Undetected		Very low
Very low birth weight	Serious limitations	No serious inconsistency	Serious indirectness	No serious imprecision	Undetected		Very low[9]
Birth asphyxia/Apgar score	No serious limitations	Serious inconsistency[10]	No serious indirectness	No serious imprecision	Undetected		Low
Hyperbilirubinaemia	No serious limitations	No serious inconsistency	No serious indirectness	No serious imprecision	Undetected	High prevalence of hearing loss	Moderate[11]
Neonatal intensive care unit (NICU) stay	Serious limitations	Serious inconsistency	Serious indirectness	No serious imprecision	Undetected		Very low[12]
Assisted ventilation	Serious limitations	Serious inconsistency	No serious indirectness	No serious imprecision	Undetected		Very low

Ototoxic drugs: aminoglycosides	No serious limitations	Serious inconsistency	No serious indirectness	No serious imprecision	Undetected		Very low[13]
Ototoxic drugs: loop diuretics	No serious limitations	Serious inconsistency	No serious indirectness	No serious imprecision	Undetected		Very low[13]
Extracorporeal membrane oxygenation (ECMO)	Serious limitations	No serious inconsistency	No serious indirectness	No serious imprecision	Undetected	High prevalence of sensorineural hearing loss	Moderate
Congenital diaphragmatic hernia	Serious limitations	Serious inconsistency	No serious indirectness	No serious imprecision	Undetected		Very low
Inhaled nitric oxide	No serious limitations	No serious inconsistency	No serious indirectness	No serious imprecision	Undetected		Very low[14]
Neurologic disease: meningitis	No serious limitations	No serious inconsistency	No serious indirectness	No serious imprecision	Undetected	High prevalence of sensorineural hearing loss	Moderate
Neurologic disease: intraventricular haemorrhage	No serious limitations	No serious inconsistency	Serious indirectness	No serious imprecision	Undetected		Very low[15]
Congenital hypothyroidism	No serious limitations	No serious inconsistency	No serious indirectness	No serious imprecision	Undetected	Population-based study	Moderate

[1] Epidemiological evidence for the risk factor for hearing loss based on rigorous observational studies/reviews

[2] Particularly late-onset hearing loss in the absence of treatment

[3] Evidence of a relationship between rubella and neonatal hearing impairment due to vaccination
[4] Few studies with strong methodology (e.g. lack of obvious information on follow-up, other risk factors not investigated, etc.); mostly case series; few patients/events
[5] Study limitations such as inaccurate information about outcome measures, absence of control group, and absence of information about other risk factors for hearing loss
[6] In most studies, confusion between the risk factors 'family history' and 'consanguinity'
[7] Owing to the high level of knowledge of genetics explaining hereditary hearing loss
[8] Despite inconsistencies among studies (few cases); one large population-based study with strong methodology
[9] Study limitations such as failure to account for confounding factors, inappropriate control group, and limited evidence of a direct relation between the risk factor and hearing loss
[10] Different cut-offs for birth asphyxia (i.e. level and timing of Apgar score) led to inconsistencies, but Apgar score at 5 minutes appears to be a good risk factor
[11] Sensitivity of the auditory system to bilirubin toxicity
[12] Methodological weaknesses such as problems in the selection of controls or sample size, confounding factors not adequately taken into account, choice of statistical analysis (i.e. univariate vs. multivariable analysis), NICU factor reflecting multiple dimensions
[13] In the context of appropriate administration of ototoxic treatments (except in cases of genetic mutation that increase susceptibility to aminoglycoside-induced hearing loss)
[14] No differences in incidence of hearing loss between groups treated with inhaled nitric oxide and oxygen
[15] Question of the role of white matter lesions over intraventricular haemorrhage on hearing loss
